# Epidemiology of cardiovascular disease and its risk factors among refugees and asylum seekers: Systematic review and meta-analysis^☆^

**DOI:** 10.1016/j.ijcrp.2022.200126

**Published:** 2022-02-10

**Authors:** Tala Al-Rousan, Rawan AlHeresh, Altaf Saadi, Hannah El-Sabrout, Megan Young, Tarik Benmarhnia, Benjamin H. Han, Laith Alshawabkeh

**Affiliations:** a Herbert Wertheim School of Public Health and Human Longevity Science, University of California San Diego, La Jolla, CA, USA; b Department of Occupational Therapy, Massachusetts General Hospital Institute of Health Professions, Boston, MA, USA; c Department of Neurology, Massachusetts General Hospital, Harvard Medical School, Boston, MA, USA; d University of California San Francisco Medical School, San Francisco, CA, USA; e Scripps Institution of Oceanography, University of California San Diego, La Jolla, CA, USA; f Division of Geriatrics, Department of Medicine, University of California San Diego, La Jolla, CA, USA; g Division of Cardiovascular Medicine, Department of Medicine, University of California San Diego, La Jolla, CA, USA

**Keywords:** Refugees, CVD, Stroke, Displaced, Migrants, Risk factors

## Abstract

**Background:**

This is the first systematic review and meta-analysis assessing cardiovascular disease incidence and risk factors among refugees and asylum seekers.

**Methods and results:**

PubMed, PsycINFO, CINAHL, and Embase databases were searched for studies in English from January 1, 1977, to March 8, 2020. Inclusion criteria were (1) observation of refugee history in participants; (2) diagnosis of CVD (coronary artery disease, heart failure, stroke, or CVD mortality) and risk factors (hypertension, diabetes, tobacco use, hyperlipidemia, obesity, psychosocial factors); (3) assessment of effect size and spread, (4) adjustment for sex; and (5) comparison with non-refugee migrants or natives. Data were extracted and evaluated by multiple reviewers for study quality. Of the 1158 screened articles, Participants from 7 studies (0.6%) involving 116.989 refugees living in Denmark, Sweden, and the United States were included in the systematic review, of which three studies synthesized the quantitative analyses. A fixed-effects model was created to pool the effect sizes of included studies. The pooled incidence of CVD in refugees was 1.71 (95% CI: 1.03, 2.83) compared with non-refugee counterparts. Pyschosocial factors were associated with increased risk of CVD in refugees but evidence on CVD risk factors varied by nativity and duration since resettlement.

**Conclusions:**

Refugee experience is an independent risk factor for CVD. Robust research on CVD in displaced populations is needed to improve the quality of evidence, clinical and preventive care, and address health equity in this marginalized population globally.

## 1 Introduction

By the end of 2020, 1 in 99 people in the world was forcibly displaced, resulting in 26.4 million refugees and four million asylum seekers, the highest number ever recorded [1]. Refugees and asylum seekers (individuals for whom their applications for refugee status are pending) are defined by the United Nations as people who have fled war, violence, conflict, or persecution and have crossed an international border to find safety in another country [[2], [3]]. A social determinant of health, migration, if forced, impacts health negatively in refugees and asylum seekers for several disease outcomes [3].

Cardiovascular disease (CVD) is the number one killer globally, accounting for one-third of deaths in 2019 [4,5]. Several studies support the association between refugee status and CVD [6,7]. A systematic review found that the risk of Ischemic heart disease in immigrants from the Middle East, the majority believed to be refugees, was 1.5 times higher than the general population of Canada [8]. Another study reported a higher risk of non-communicable diseases, including CVD, among refugees [7].

A growing body of literature challenges the “healthy migrant effect” hypothesis revealing health disparities related to distinct exposures and life experiences of refugees and asylum seekers [9]. For example, interrupted medical care and preventive care along the migratory route and post-resettlement [10], psychosocial stressors, racism, and history of living in migrant or refugee camps where the average stay in a refugee camp is seventeen years each negatively impact health [11]. On the other hand, refugees' resilience, agency, and coping methods with traumatic events may moderate these causal relationships between forced migration and disease outcomes [12].

Although several risk factors for CVD are prevalent in refugees [[13], [14], [15]], [[13], [14], [15]] and there is evidence on the increased CVD prevalence and risk in migrants in general in some studies, the extent to which the risk of CVD is specifically increased among refugees and asylum seekers remains unclear. To date, no comprehensive synthesis of CVD risk in refugees has been conducted. Therefore, this study aims to compare CVD risk factors and diagnoses between refugees and native populations through a systematic review and a meta-analysis.

## 2 Methods

This study is a systematic review of the literature and a meta-analysis registered in PROSPERO (No. CRD42020201271). The Preferred Reporting Items for Systematic Reviews and Meta-analysis (PRISMA) [16] and the Meta-analysis of Observational Studies in Epidemiology (MOOSE) were used to guide this review.

### 2.1 Search strategy

A literature search was conducted on August 17, 2020 (Appendix A.), using PubMed, EMBASE, PsycINFO, and CINAHL databases. Inclusion criteria were: published in an English peer-reviewed journal, confirmed refugee status, reported a CVD diagnosis (physician report, self-report, mortality), CVD incidence that was at least adjusted for one variable at a minimum (i.e., sex), and a comparator group that was a native population or non-refugee migrants. Studies were excluded if an explicit description of the refugee status was not provided or if the study included children only. Appendix A includes the electronic database search queries. The search was complemented using a hand search, and authors of studies were contacted if needed. The search did not have any restrictions on publication date. Search output was organized using Covidence ®, a web-based tool used for systematic collaborative reviews. One author completed the search and importing of data into the online tool. Duplicates of study titles from merging database outputs were removed.

### 2.2 Study selection, data collection, and data extraction

Two reviewers (TA, RA) independently reviewed the search output for eligibility (titles, abstracts, then full text). A third reviewer was consulted (AS) to resolve disagreements. For included studies, data extraction included author/year of publication, country of study, origin or ethnicity of refugees, number of refugees with CVD, observation period, data source, the effect size of incidence of CVD, and their confidence intervals. If found in those articles, risk factors were also extracted, including tobacco use, substance abuse, psychosocial factors, obesity, CVD, hypertension, diabetes, and high cholesterol but were not the primary outcome of interest for this study.

### 2.3 Quality of the included studies

The quality of the studies was assessed independently by two reviewers (HE, MY) using the Crow Critical Appraisal Tool (CCAT), an established critical appraisal tool with established construct validity and reliability [[17], [18], [19]]. Using this tool, the quality appraisal is reviewed in eight categories: preliminary appraisals, introductions, design, sampling procedures, data collection methods, ethical considerations, results, and discussions. Each category is scored out of 5 points (total score ranging from 0 to 40). A CCAT checklist was completed for each included study, and a total score was assigned to reflect the quality, with higher scores reporting higher quality.

### 2.4 Outcomes

The primary outcomes were CVD incidence (including only nonfatal or a combination of nonfatal and fatal outcomes of a composite of different CVD outcomes). Secondary outcomes included incidence of nonfatal or a combination of nonfatal and fatal outcomes, and mortality from fatal outcomes (i.e., coronary heart disease (CHD), stroke, Atrial Fibrillation (A.F), and Heart failure (H·F)).

Studies that reported fatal CHD and nonfatal myocardial infarction separately were combined using a fixed-effects model to generate an overall estimate for CHD incidence. In the same way, following the same procedure, ischemic stroke outcomes and fatal ischemic stroke and fatal hemorrhagic stroke endpoints were combined to obtain an overall estimate for stroke incidence and stroke mortality [20,21], respectively.

### 2.5 Statistical analysis and data synthesis

The primary outcome was the pooled relative risk and 95% confidence intervals (CI) of CVD diagnosis and risks in refugees compared to non-refugee migrants and native populations. For our main analysis, we conducted a fixed-effects meta-analysis to derive a pooled relative risk and 95% CI representing the differential CVD outcome [22,23]. We also conducted a random-effect meta-analysis as sensitivity analysis (see supplementary materials Appendix C). We have only included CVD incidence in the meta-analysis and not prevalence since the latter is likely prone to selection bias and regression to the mean. Results of the meta-analysis were reported results using forest plots. Heterogeneity across studies was assessed using the I^2^ statistic [23], where larger values of I^2^ represent more significant heterogeneity in the relative risk estimates across studies. We conducted Egger's test for small-study effects and examined funnel plots to identify publication bias. Finally, we implemented a leave-one-study-out analysis to assess how our pooled estimates were driven by one single study (see supplementary materials Appendix C).

## 3 Results

**Fig. 1:**
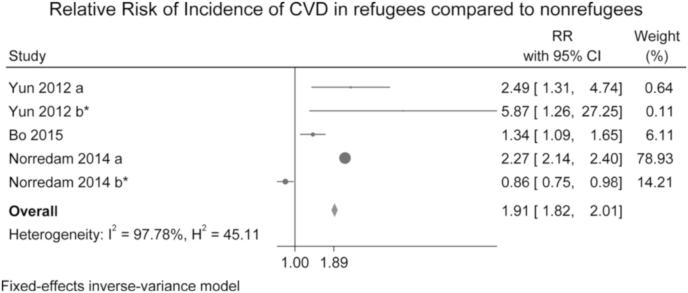
Relative Risk of Incidence of CVD in refugees compared to nonrefugees

Fig. 1 presents the PRISMA flowchart of study selection procedures. The search yielded 738 study titles from Pubmed, 385 study titles from EMBASE, 22 from PsychINFO, and 376 from CINHAL databases. After duplicates removal, 1,133 study titles remained; 1,041 were omitted for not meeting inclusion criteria. Of those, ninety-one full-text studies were reviewed, and seven met all the inclusion criteria for the systematic review, of which three were used in the meta-analysis because of the inclusion of necessary data to calculate an R.R.

The seven included studies present data on 479,361 refugees collected between 1993 and 2016 in the US, Denmark, and the Netherlands. Data sources used in these studies include registries and electronic medical records. The quality scores of the studies ranged between 23 and 36, averaging 28/40, indicating a moderate quality of the literature (Table 1.).

**Table 1:** Characteristics of included studies in the literature review of cardiovascular disease incidence in refugees and asylum seekers.

Authors (Year)	Country/Region of native population	Origin or ethnicity of Refugees	N of refugees and asylum seekers	% of women	Observation period	Study type	Data source	Outcome	Confounders adjusted for	Quality score
Bo et al. (2015)	Denmark	Afghanistan, Iraq, Somalia, Turkey, East Asia and the Pacific, Eastern Europe and Central Asia, South Asia, South and middle America, Sub-Saharan Africa, The Former Yugoslavia, The Middle East, and North Africa	113,918	28.2	1993–1999	Historical prospective cohort	Danish National Patient Registry, the National Causes of Death Registry, Registry for Population Statistics	First-time CHD incidence	Age, income	35/40
Linton et al. (2020)	USA	Burma; Bhutan; Moldova; Russia; Somalia; Ukraine	30,243	47.1	2006–2016	Retrospective cohort	Worldwide Refugee Admissions Processing System	Cause of death	Age, region of origin	23/40
Nguyen et al. (2016)	USA	Iran, Ukraine, Vietnam	21,968	51.7	2002–2011	Cross-sectional	The California Department of Public Health's Refugee Health Electronic Information System	Prevalence of chronic conditions	Age, sex	34/40
Njeru et al. (2016)	USA	Somalia	1007	61.1	2008–2012	Retrospective Cohort	Electronic Medical Records	Prevalence of CVD	Age, sex, Body Mass Index (BMI), education, employment, time since first clinic visit	35/40
Norredam et al. (2014)	Denmark	East Europe, Baltics, and Central Asia Former Yugoslavia Western Asia and North Africa, including Turkey and Iraq Sub-Saharan Africa South-Eastern and Eastern Asia South Asia Western Europe, the Americas, Australia, and New Zealand	43,992	41.6	1993–2010	Historical prospective cohort	Personal ID number cross-references with Danish National Patient Register	First-time CVD incidence	Age, sex	35/40
van Oostrum et al. (2011)	Netherlands	WCS Africa NEH Africa CES Europe ME/SW Asia CES Asia Stateless persons, South and Central America	222,217	39.5	2002–2005	Historical prospective cohort	Community Health Services for Asylum Seekers	Cause of death	Age, country of origin	36/40
Yun et al. (2012)	USA	East, South, and Southeast Asia Europe and Central Asia Latin America and the Caribbean The Middle East and North Africa Oceania and Canada Sub-Saharan Africa	490	50.1	2003	Cross-sectional	2003 New Immigrant Survey (NIS)	Prevalence of chronic conditions	Age, time in the United States, sex, region of origin, marital status, education, English proficiency, employment status, and income	34/40

### 3.1 Meta-analysis results

**Fig. 2:**
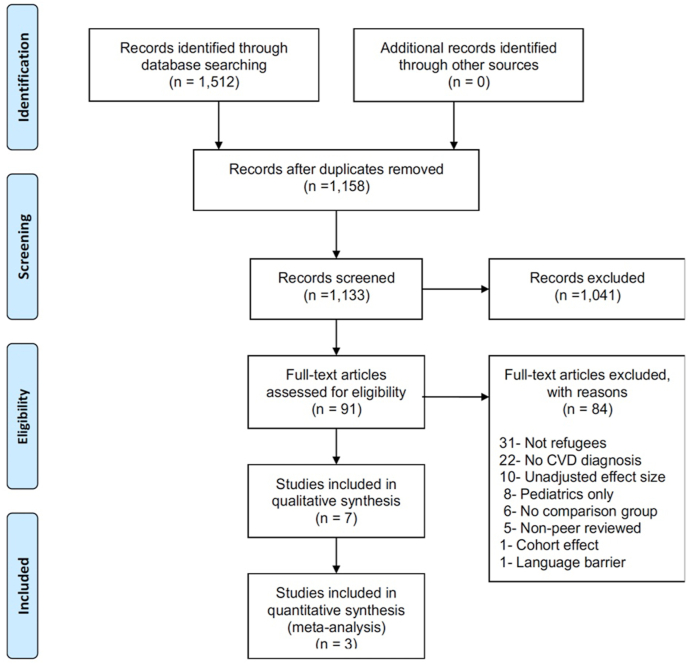
PRISMA flow chart of study selection in systematic review and meta-analysis.

Three studies were included in the meta-analysis, with two studies reporting two estimates of relative risk (RR) of CVD per study [20,24] to have a total of 5 estimates pooled for RR (Fig. 2). The pooled RR from the fixed-effects meta-analysis was 1.91 (95% CI: 1.82, 2.01) compared to native or non-refugee migrants. Heterogeneity among studies was high (I^2^ 97.8%). When using random-effects, we found a pooled RR of 1.71 (95% CI: 1.03, 2.83) (see supplementary materials Appendix C). All except one study depict a consistent positive RR highlighting that refugees had a higher probability of CVD diagnosis. We conducted an Egger's test for small-study effects and found a non-significant bias (*p* = 0.12). Only two studies [20,24] reported stroke as an outcome. When removing these two studies from the meta-analysis, which was determined by the investigators a priori, the pooled RR was 1.89 (95%CI: 1.27, 2.81). Risk Factors of CVD are reported in Table 2. All analyses were conducted with Stata Statistical Software: Release 16 SE. College Station, TX (StataCorp LLC).

**Table 2:** Summary of reported cardiovascular disease risk factors in refugees and asylum seekers.

Authors (Year)	D.M.	Hyperlipidemia	Hypertension	Obesity	Psychosocial	Tobacco
Bo et al. (2015) [25]	NR	NR	NR	NR	NR	NR
Linton et al. (2020) [34]	NR	NR	NR	NR	NR	NR
Nguyen et al. (2016) [22]	Iran (↓)	NR	Iran (↓)	Iran (↑)	NR	Iran (↑) Ukraine (↓) Vietnam (↑)
	Ukraine (↓)		Ukraine (↓)	Ukraine (↑)		
	Vietnam (↓)		Vietnam (↓)	Vietnam (↓)		
Njeru et al. (2016) [23]	↑	↓	NS	↑	NR	NR
Norredam et al. (2014) [20]	↑	NR	NR	NR	NR	NR
van Oostrum et al. (2011) [24]	NR	NR	NR	NR	↑	NR
Yun et al. (2012) [21]	NS	NR	↑	NR	↑	NR

↑ increased odds; ↓ decreased odds; ↔ not significant; N.R. not reported.

#### 3.1.1 Diabetes mellitus (D.M.)

Of the seven studies included, four examined D.M. prevalence in refugees [20,[24], [25], [26]]. When compared with native populations, refugees demonstrated significantly higher rates of D.M. in two studies [24,26]. This difference was significant within 18 years of follow-up [24]. Although one study found higher D.M. prevalence in the refugee population; the results were not statistically significant compared to an immigrant comparator group [20]. The remaining study found a lower prevalence of D.M. in refugees than the native population that trended upwards with age [25]. However, this study looked at only recent arrivals using the state department of public health electronic health information system. It is established in the literature that recent arrivals of immigrants are usually healthier than the general population, but this observation is reversed over time.

#### 3.1.2 Hyperlipidemia (HDL)

Only one study reported hyperlipidemia status, where hyperlipidemia was lower among refugees than native populations [26]. This study of Somali refugees did not report on years since resettlement and relied on chart reviews of patients within five years.

#### 3.1.3 Hypertension (HTN)

In one study, refugees were more likely to have hypertension than non-refugee immigrants, with hypertension increasing with age in all groups [20]. This study had a large sample size and included refugees living in the US for longer than 1 year, which can help reduce the “health migrant effect” bias. However, it has utilized the 2003 New Immigrant Survey. Another study that examined hypertension prevalence within three separate refugee groups, Iran, Ukraine, and Vietnam, found hypertension lower in refugees when compared to the native population, but this study looked at only recent arrivals of less than 90 days as examined in initial screenings [25]. One study did not find hypertension prevalence significantly different between Somali refugees and native populations, but this was a study from chart reviews of only one hospital and did not collect data on time since arrival [26].

#### 3.1.4 Tobacco use

Only one study assessed tobacco use [25]. It showed that the Iranian and Vietnamese refugee groups had a higher prevalence of tobacco use than native populations, while Ukrainian refugees had a lower prevalence. This study is of recent arrivals only and is not a true reflection of post-resettlement tobacco use in refugees over time. Tobacco use remains prevalent in countries producing refugees worldwide, yet behaviors related to tobacco and other substance use in refugees post resettlement are lacking.

### 3.2 Obesity

Two studies compared the prevalence of obesity in refugees with that of native populations [25,26]. One study found a higher prevalence in Somali refugees who have been living in the US for some time [26]. The other study compared three refugee groups from Iran, Ukraine, and Vietnam to the native population and found a higher prevalence of obesity in Iranian and Ukrainian refugees than native populations [25].

### 3.3 Psychosocial factors

Two of the included studies evaluated psychosocial factors [20,27]. Compared to non-refugee migrants, refugees reported significantly higher rates of behavioral health problems [20]. Male refugees had an increased rate of mortality from mental and behavioral disorders [27].

## 4 Discussion

We report the first comprehensive systematic review and meta-analysis comparing CVD risk factors in refugees to non-refugee migrants and native populations. This meta-analysis indicates that refugee status is associated with an increased risk of CVD compared to non-refugee counterparts. Our systematic review yielded a paucity of studies that directly compared CVD risk factors in refugees to other migrant and host communities, which underscored a research gap and missed opportunity to combat the CVD burden globally, given that the number of refugees increases is at an all-time high. Additionally, most studies on risk factors relied on secondary data analysis of electronic health records either in one hospital or using the state system of new arrivals of refugees. Only one primary data collection effort through the 2003 New American Survey allowed for a representative sample of refugees reporting on CVD risk factors [20].

Studies included in our analyses were comparable in methods used, countries of origin, and CVD risk outcomes. For example, Norredam et al. reported CVD outcomes, including stroke and ischemic heart disease, and Yun et al. [20,24] reported heart disease as a general category and stroke. In addition, the Danish-based studies included used registry-based cohorts linked to the Danish Immigration Service, where refugee status was ascertained post resettlement. In contrast, the one U.S.-based study used a nationally representative sample where participants self-reported their refugee status and CVD diagnoses.

All, except one study, observed an increased risk of CVD in refugees than non-refugee migrants or native populations. The Danish studies were the only ones that compared refugees to native populations and non-refugee migrants, while the U.S.-based study compared the risk of CVD in refugees to non-refugee migrants only. The higher CVD risk in refugees indicates that refugees are a distinct group of migrants with unique experiences and exposures that may portend different risk patterns than migrants originating from the same country.

Bo et al. reported the lowest risk of CVD in refugees [28]. A potential reason for this is that the study was a retrospective cohort study that reported incident CVD. In contrast, the other two studies by Norredam et al. and Yun et al. compared CVD prevalence to obtain R.R.

Refugees originate from all over the world, and understanding whether refugees from certain parts of the world are at higher risk of CVD remains unclear. Country of origin was not consistently listed. Bo et al. were the only ones to compare CVD risk by country of origin and showed that refugees from the Middle East reported the highest CVD risk [28]. However, it is unclear why the authors did not include Iraqi and Afghani refugees within the Middle Eastern refugees' category, which may have underestimated CVD risk in the Middle eastern refugee group. This finding is consistent with another review that examined Middle Eastern refugees without directly comparing them to native populations or CVD rates in countries of origin [29]. Findings focusing on the region of origin are significant considering a recent report of global patterns of CVD, which showcased the Middle East region to have one of the highest age-standardized disability-adjusted-life-years (DALY) rates due to ischemic heart disease, and one of the highest numbers of stroke survivors globally [8]. However, the question remains whether the CVD profile of refugees resembles that of the countries where they are coming from or if the refugee experience itself may increase one's risk of CVD. Research on risk accumulated before, during, and after resettlement is needed in different migrant groups to characterize CVD risk better.

The association of specific demographics such as sex, country of origin, and length of stay with CVD risk in refugees remains understudied. CVD risk by sex was only reported by Bo et al. Interestingly, both refugee men and women had a higher incidence of CVD than other immigrants from the same countries of origin when analyzing country of birth and migration status simultaneously. However, compared to native populations, only refugees from certain countries had a higher CVD risk by sex. This can be explained by the differing refugee resettlement processes and requirements, which allow for selecting the healthiest individuals and migration. Norredam et al. reported that the length of stay was negatively associated with worse CVD outcomes in refugees over time [24].

Only three studies had a low risk of bias. Future research must be of high rigor and reporting quality to determine precise risk estimates. While reviewing studies through the inclusion process, we identified methodological practices known to be erroneous that we think should be avoided in the future. First, the selection of confounders in included studies was rarely justified, and in most studies, only a few covariates (e.g., age and sex) were included. Identifying confounders of interest in the relationship between refugee status and CVD risk is critical to obtain valid effect estimates. Various approaches exist to identify such variables, such as Directed Acyclic Graphs [[30], [31], [32]] that help identify variables that need to be controlled to limit confounding and increase the transparency of analytical choices. For example, some covariates related to CVD risk factors that would be concomitantly induced by refugee status (like hypertension or psychosocial factors) are mediators and should not be controlled. Second, we found that many studies reported (and interpreted) regression coefficients for all included variables (e.g., age) while the primary exposure of interest was refugee status. Such practices, also known as the “Table 2 fallacy,” [33] have been identified as a source of confusion in other areas of refugee research. Reporting (and interpreting) coefficients could prevent this for the primary exposure of interest (i.e., refugee status). Future refugee health research must consider addressing this early in the study design process. (Appendix B).

Additionally, the underlying mechanisms explaining the increased CVD risk in refugees have been investigated in some of the studies included, but more research is needed to explain these risk patterns. Yun et al. adjusted for sociodemographic variables, including English language proficiency, age, sex, and health insurance coverage. Norredam et al. adjusted for only age and sex, while Bo et al. found income to be a potential explanation for the increased CVD risk in refugees. Caution must be exercised when adjusting for income since it depends on the time of refugee arrival, naturalization, and access to jobs. Recent research has examined socioeconomic disadvantage and found that neighborhood characteristics, including income, unemployment, crime rate, welfare participation, and education, were individually and collectively associated with increased CVD risk, especially in young refugee populations [34].

Moreover, uncontrolled risk factors may explain these patterns, highlighting the need for health promotion and preventive services tailored towards refugees and asylum seekers. A prevalence study in a U.S. refugee clinic found that although refugees had comparable prevalence rates of CVD, uncontrolled hypertension and diabetes rates were higher, at 46.7% and 49.2%, respectively [35]. More than a third of refugees were diagnosed with hypertension, a quarter with hyperlipidemia, and a significant proportion with D.M, myocardial infarction, and stroke. Rates of all conditions except stroke were higher among refugees compared with native-born Danes matched by age and sex [34]. Other possible mediators may include stress (a central construct in refugee experiences), health behaviors (i.e., tobacco use), and mental health (i.e., post-traumatic disorders), all prevalent in refugees. Further research on the biological plausibility of these hypotheses is needed and will have significant implications for CVD prevention and clinical management plans for refugees. Future longitudinal research accounting for migration-specific characteristics, including pre, during, and post-migratory variables including access to care and length of stay in the resettlement country, are needed to establish causality and quantify risk.

Although this is the first systematic review and meta-analysis on CVD risk among refugees, the generalizability and validity of our study are limited. The included studies all come from either Denmark or the U.S, which hinders generalizing the results to other contexts where resettlement processes, integration within new communities, and characteristics of native populations vary. Although methodologically not comparable, this Dutch study found that mortality among refugees younger than 40 was higher than the Dutch population, with CVD being the most common cause of mortality [36]. Second, surveys and registry-based studies were included in this analysis which offers large sample sizes and reduced selection bias in using patient registries. Nevertheless, refugees not officially registered as such or who are not engaged in or able to afford healthcare may have worse CVD risk profiles since most refugees face socioeconomic difficulties [37,38]. Third, some of the included studies did not consistently adjust for many potential confounders, such as socioeconomic status. The lack of adjustment for confounding variables may have contributed to the overestimation of the effect size. Our study demonstrated moderate methodologic rigor within a limited body of evidence examining forced displacement on cardiovascular health. There have been very few data sets collecting data on refugee history in the US, which hampers conducting epidemiological research in this field [39]. One additional limitation is that some studies included native population in the comparison group which is not directly comparable with studies that used non-refugee immigrant only as the comparison group. Finally, only studies published in English were included, such that some valuable studies published in other languages would have been missed. Given that most of the world's refugee population resides in low- and middle-income countries where the association between low socioeconomic status and CVD incidence and CVD mortality are high [40], research endeavors and infrastructure must be strengthened to study refugees in these countries. Future studies need to further explore differences in risk between different referent groups beyond native populations, including non-refugee immigrants who may have different exposures to trauma alongside different pathways to citizenship and healthcare access but similar post-migration experiences such as language barriers or acculturative stress.

## 5 Conclusion

The present systematic review and meta-analysis provide the most updated and comprehensive summary estimates of the association between forced displacement and CVD outcomes. Although a significant threat to global public health, forced displacement is a social determinant of many health outcomes, including understudied cardiovascular health. In this analysis, refugee history was associated with an increased risk of CVD and must be considered an independent risk factor targeted by CVD prevention efforts. Resettlement policies must expand their focus from initial health screening post-arrival on infectious diseases to non-communicable diseases including CVD using universal and agreed-upon screening methods by the global community. Renewed focus and commitment are essential for providing affordable, accessible, and evidence-based implementation strategies to effectively prevent, treat, and manage CVD in the diverse displaced populations.

## Credit author statement

**Tala Al-Rousan**: Conceptualization, methodology, manuscript draft preparation, **Rawan Al-Heresh**: Conceptualization, methodology, manuscript draft preparation, **Altaf Saadi**: Methodology, data analysis, **Hannah El-Sabrout**: Data analysis, **Megan Young**: Data analysis, **Tarik Benmarhnia**: Data analysis, interpretation of results, manuscript draft preparation, **Benjmain Han**: Data analysis, interpretation of results, **Laith Alshawabkeh**: Methodology, interpretation of study results, writing, reviewing and editing.

## Footnotes

Appendix A Supplementary data to this article can be found online at https://doi.org/10.1016/j.ijcrp.2022.200126.
