# Supplementary material for: Epidemiology of cardiovascular disease and its risk factors among refugees and asylum seekers: Systematic review and meta-analysis
Source: Int J Cardiol Cardiovasc Risk Prev. 2022 Feb 10;12:200126. doi: 10.1016/j.ijcrp.2022.200126 (PMC8851152; doi:10.1016/j.ijcrp.2022.200126)
Supplement: Multimedia component 1 [file mmc1.docx]

**Appendix A. Search queries**

***Search date: August 17^th^ 2020***

Pubmed: Search yielded 738 papers

EMBASE: Search yielded 385 papers

PsychINFO: Search yielded 22 papers

CINHAL: Search yielded 367 papers

Total: 1,512 titles

After Duplicate removal: 1,158 titles

PubMed Query:

Search: (((((((Refugee) OR (Asylum seeker)) OR (migrant)) OR (forced migration)) OR (persecut)) OR (immigrant and torture)) AND (CardioVascular Disease[MeSH Terms])

((((((("refugee s"[All Fields] OR "refugees"[MeSH Terms]) OR "refugees"[All Fields]) OR "refugee"[All Fields]) OR ((("refugees"[MeSH Terms] OR "refugees"[All Fields]) OR ("asylum"[All Fields] AND "seeker"[All Fields])) OR "asylum seeker"[All Fields])) OR ((((("migrant s"[All Fields] OR "transients and migrants"[MeSH Terms]) OR ("transients"[All Fields] AND "migrants"[All Fields])) OR "transients and migrants"[All Fields]) OR "migrant"[All Fields]) OR "migrants"[All Fields])) OR ("forced"[All Fields] AND (((((((("migrate"[All Fields] OR "migrated"[All Fields]) OR "migrates"[All Fields]) OR "migrating"[All Fields]) OR "migration"[All Fields]) OR "migrational"[All Fields]) OR "migrations"[All Fields]) OR "migrator"[All Fields]) OR "migrators"[All Fields]))) OR ((((((((((((((("emigrants and immigrants"[MeSH Terms] OR ("emigrants"[All Fields] AND "immigrants"[All Fields])) OR "emigrants and immigrants"[All Fields]) OR "immigrant"[All Fields]) OR "immigrants"[All Fields]) OR "emigration and immigration"[MeSH Terms]) OR ("emigration"[All Fields] AND "immigration"[All Fields])) OR "emigration and immigration"[All Fields]) OR "immigration"[All Fields]) OR "immigrations"[All Fields]) OR "immigrant s"[All Fields]) OR "immigrate"[All Fields]) OR "immigrated"[All Fields]) OR "immigrates"[All Fields]) OR "immigrating"[All Fields]) AND (((((("torture"[MeSH Terms] OR "torture"[All Fields]) OR "tortures"[All Fields]) OR "tortured"[All Fields]) OR "torturer"[All Fields]) OR "torturers"[All Fields]) OR "torturing"[All Fields]))) AND "cardiovascular diseases"[MeSH Terms]

Translations

**Refugee:** "refugee's"[All Fields] OR "refugees"[MeSH Terms] OR "refugees"[All Fields] OR "refugee"[All Fields]

**Asylum seeker:** "refugees"[MeSH Terms] OR "refugees"[All Fields] OR ("asylum"[All Fields] AND "seeker"[All Fields]) OR "asylum seeker"[All Fields]

**migrant:** "migrant's"[All Fields] OR "transients and migrants"[MeSH Terms] OR ("transients"[All Fields] AND "migrants"[All Fields]) OR "transients and migrants"[All Fields] OR "migrant"[All Fields] OR **"migrants"[All Fields]**

**migration:** "migrate"[All Fields] OR "migrated"[All Fields] OR "migrates"[All Fields] OR "migrating"[All Fields] OR "migration"[All Fields] OR "migrational"[All Fields] OR "migrations"[All Fields] OR "migrator"[All Fields] OR "migrators"[All Fields]

**immigrant**: "emigrants and immigrants"[MeSH Terms] OR ("emigrants"[All Fields] AND "immigrants"[All Fields]) OR "emigrants and immigrants"[All Fields] OR "immigrant"[All Fields] OR "immigrants"[All Fields] OR "emigration and immigration"[MeSH Terms] OR ("emigration"[All Fields] AND "immigration"[All Fields]) OR "emigration and immigration"[All Fields] OR "immigration"[All Fields] OR "immigrations"[All Fields] OR

"immigrant's"[All Fields] OR "immigrate"[All Fields] OR "immigrated"[All Fields] OR "immigrates"[All Fields] OR "immigrating"[All Fields] OR "immigration's"[All Fields]

**torture:** "torture"[MeSH Terms] OR "torture"[All Fields] OR "tortures"[All Fields] OR "tortured"[All Fields] OR "torturer"[All Fields] OR "torturers"[All Fields] OR "torturing"[All Fields]

**CardioVascular Disease [MeSH Terms]:** "cardiovascular diseases"[MeSH Terms]

EMBASE query:

Query: 'cardiovascular disease'/exp AND 'refugee'/exp

PsychINFO query:

Database(s): **APA PsycInfo**1967 to August Week 2 2020

Search Strategy:

| **#** | **Searches** | **Results** |
| --- | --- | --- |
| 1 | exp Cardiovascular Disorders/ | 62099 |
| 2 | exp Refugees/ | 6183 |
| 3 | 1 and 2 | 22 |

CINHAL query:

| **Search ID#** | **Search Terms** | **Search Options** | **Last Run Via** | **Results** |
| --- | --- | --- | --- | --- |
| S3 | S1 AND S2 | **Expanders** - Apply equivalent subjects **Search modes** - Boolean/Phrase | **Interface** - EBSCOhost Research Databases **Search Screen** - Advanced Search **Database** - CINAHL Complete | 387 |
| S2 | S.U. refugees or asylum seekers or displaced or migrants or immigrants or emmigration | **Expanders** - Apply equivalent subjects **Search modes** - Boolean/Phrase | **Interface** - EBSCOhost Research Databases **Search Screen** - Advanced Search **Database** - CINAHL Complete | 26,012 |
| S1 | AB cardiovascular disease or cvd or heart or cardiac or coronary heart disease | **Expanders** - Apply equivalent subjects **Search modes** - Boolean/Phrase | **Interface** - EBSCOhost Research Databases **Search Screen** - Advanced Search **Database** - CINAHL Complete |  |

**Appendix B: Illustrative example of the Table 2 fallacy**

To answer the etiological research question as an example: “what is the effect of refugee history on the risk of stroke?”, we can consider a binary exposure (refugee status, yes or no) in a specific study population (that we assume to be representative of the target population of interest) and incident stroke (yes/no) as the main outcome of interest (for simplicity, focusing on the cumulative incidence of stroke). 2 confounders are assumed to affect this relationship: age and gender. This is represented in the directed acyclic graph (DAG) below.


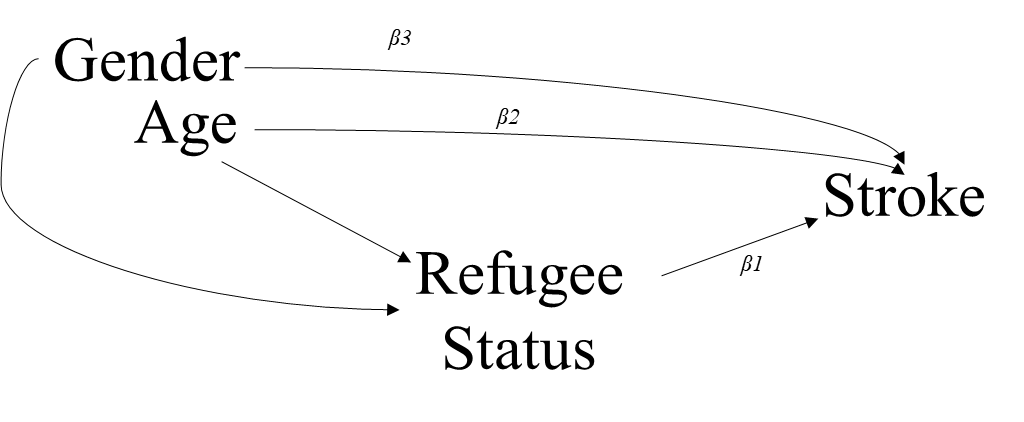


In this setting, a multivariable logistic regression model where stroke is the dependent variable and refugee status, age and gender are included as independent variables with a coefficient for each of these three independent variables assumed. The exponential of the coefficient (*β1*) for refugee status is an odds ratio (OR). We assume that the DAG is correct, the outcome is rare, no selection bias, no misclassification and correct model specification. This OR will approximate our causal quantity of interest and can be thus interpreted as the effect of refugee status on the risk of stroke. However, in this setting the interpretation of the coefficients for age (*β2*) and gender (*β3*) is quite different. Indeed, refugee status is considered as an intermediate (mediator) between age/gender and stroke and the coefficients for both age and gender represent the association between each of these 2 variables and stroke removing the pathway through refugee status. Such interpretation is therefore not directly related to the main question of interest which can lead to some confusion especially in the context of systematic reviews or meta-analyses. If the quantification of the effect of age, gender or any other variable on the risk of stroke is of interest, a dedicated DAG (with identified confounders for a specific exposure of interest) is required as well as a separate analysis. This is the reason for which we recommend to only present and interpret coefficients related to the main exposure of interest for such inferential research questions.
